# Engineered Adipose-Derived Stem Cells Overexpressing RXFP1 via CRISPR Activation Ameliorate Erectile Dysfunction in Diabetic Rats

**DOI:** 10.3390/antiox12010171

**Published:** 2023-01-11

**Authors:** Taotao Sun, Wenchao Xu, Bocheng Tu, Tao Wang, Jihong Liu, Kang Liu, Yang Luan

**Affiliations:** 1 Department of Urology, Tongji Hospital, Tongji Medical College, Huazhong University of Science and Technology, Wuhan 430030, China; 2 Institute of Urology, Tongji Hospital, Tongji Medical College, Huazhong University of Science and Technology, Wuhan 430030, China

**Keywords:** adipose-derived stem cells, RXFP1, CRISPR activation, oxidative stress, erectile dysfunction, diabetes mellitus

## Abstract

Due to the high incidence of diabetes mellitus (DM) and poor response to the first-line treatment of DM-induced erectile dysfunction (DMED), new therapeutic strategies for DMED are needed. Adipose-derived stem cell (ADSC) transplantation is considered a promising treatment modality for DMED but is limited by poor survival and efficacy after transplantation. In this study, we aimed to increase the therapeutic effect of DMED by overexpressing the relaxin family peptide receptor 1 (RXFP1) using a clustered regularly interspaced short palindromic repeats activation (CRISPRa) system in ADSCs. Two lentiviruses carrying the CRISPRa system transfected ADSCs to overexpress RXFP1 (RXFP1-ADSCs). The intracavernous injection of ADSCs was performed in DMED rats induced by the intraperitoneal injection of streptozotocin. Four weeks after transplantation, we measured erectile function and collected specimens of the corpus cavernosum for follow-up detection. The results showed that ADSCs improved erectile function in diabetic rats, and the RXFP1-ADSCs were more significant. We detected reduced levels of oxidative stress, apoptosis and fibrosis together with relative normalization of endothelial and smooth muscle cell function in the penis after ADSC transplantation. RXFP1-ADSCs had more potent efficacy in the above alterations compared to negative control ADSCs due to the high levels of survival and paracrine capacity in RXFP1-ADSCs. The results revealed that RXFP1-ADSC transplantation could partially preserve erectile function in DMED rats associated with the regulation of oxidative stress, apoptosis, fibrosis and endothelial and smooth muscle cell dysfunction. RXFP1 may be the new target for the genetic modification of ADSCs, which benefits the management of DMED.

## 1. Introduction

Erectile dysfunction (ED) is one of the common afflictions in male sexual dysfunction, which refers to the inability of the penis to be hard enough for satisfactory sex. The etiology of ED is complex, including aging, nerve damage, drugs, various metabolic abnormalities and other factors [1]. Hypertension, hyperlipidemia, obesity, metabolic syndrome and other metabolic abnormalities can lead to the production of ED or exacerbate the process of ED. Given the large number of patients with diabetes mellitus (DM), DM-induced ED (DMED) has become an area that cannot be ignored in the field of ED. The latest data from the International Diabetes Federation shows that more than 10.5% of adults worldwide suffer from diabetes [2]. In addition, phosphodiesterase type 5 inhibitors (PDE5is) are widely used as a first-line treatment for ED. However, the effective rate of PDE5is is significantly reduced in patients with DMED compared to non-diabetic patients (63% vs. 83%) [3]. Therefore, new therapeutic strategies need to be explored to achieve better therapeutic effects in DMED patients.

Mesenchymal stem cells are adult stem cells with multi-lineage differentiation ability. Intracavernous injection of mesenchymal stem cells is currently considered one of the most promising options in treating ED. Several animal and clinical studies have demonstrated the efficacy of mesenchymal stem cells in ED [4,5]. Compared with bone-marrow-derived stem cells (BMSCs) and skeletal-muscle-derived stem cells, adipose-derived stem cells (ADSCs) have attracted more attention due to the advantages of abundant in vivo content, simple extraction, easy isolation and culture and low immunogenicity [6]. However, the survival and efficacy of ADSCs after transplantation are limited due to the harsh pathological environment in recipients, such as oxidative stress induced by hyperglycemia [7,8]. One of the solutions is to genetically modify ADSCs to increase the quality and quantity of ADSCs after transplantation, which has significantly improved the application value of ADSCs in DMED [9].

Relaxin is an endocrine hormone discovered in 1926 to relax the birth canal and is also known as an insulin-like peptide because of its similar structure to insulin. Relaxin-2 (RLX-2) is the predominant type of relaxin in human circulation and has attracted much attention due to its various cardiovascular protective functions [10]. As the major receptor for relaxin-2, the relaxin family peptide receptor 1 (RXFP1) is a transmembrane G-protein-coupled receptor that is widely present in the cardiovascular system, reproductive system, kidney, lung, liver and other organs [11]. Multiple studies have demonstrated that RXFP1 can regulate the function of mesenchymal stem cells, endothelial progenitor cells and adult cells, enhancing their therapeutic applications [12,13,14]. Previously, we demonstrated that RLX-2 improved erectile function in rats with bilateral cavernous nerve injury via RXFP1 [15]. However, it remains unclear whether RXFP1 could improve the therapeutic effect after ADSC transplantation in DMED.

The clustered regularly interspaced short palindromic repeats (CRISPR) system is a powerful, customizable and RNA-guided genome editing tool that consists of single guide RNA (sgRNA) and CRISPR-associated protein 9 (Cas9) [16,17]. In recent years, new editing technologies have been explored based on CRISPR. The fusion of catalytically inactive Cas9 (dCas9) with a transcriptional activator (e.g., VP64), combined with the guidance of sgRNA, can upregulate the expression of target genes, which is called CRISPR activation (CRISPRa) [18]. CRISPRa has been used with promising results in various fields, including stem cell engineering and regenerative medicine [19]. Unfortunately, there is no research to verify the efficacy of CRISPRa in the field of ED.

In this study, we first verified the expression of RXFP1 in ADSCs and genetically modified ADSCs to activate the expression of RXFP1 (RXFP1-ADSCs) via CRISPRa. Next, the type I diabetic model of rats was constructed using STZ and received intracavernous injections of negative control (NC) ADSCs and RXFP1-ADSCs. Finally, we compared the differences in treatment in different groups of rats and explored the possible underlying mechanisms.

## 2. Materials and Methods

### 2.1. Culture and Identification of Cells

ADSCs were primarily isolated from the inguinal fat pad of Sprague Dawley rats. The cells were suspended in Dulbecco’s modified Eagle’s medium (DMEM, Boster, Wuhan, China) supplemented with 10% fetal bovine serum (FBS; GIBCO, Grand Island, NY, USA) and cultured in a suitable environment (5% CO_2_, 37 °C).

After 4 passages, flow cytometry was carried out to identify ADSCs. CD29, CD31, CD34, CD45, CD90 and CD106 were chosen to detect cell surface markers [20]. The multi-lineage differentiation ability of ADSCs, including adipogenic, endothelial and smooth muscle differentiation, was examined using inducing differentiation media [20,21,22,23]. Oil-red-O staining and immunofluorescence (anti-vWF and anti-α-SMA) were applied for the identification of the final result. The details of the antibodies used are listed in Table S1.

### 2.2. Transfection of Cells

The reference sequence of RXFP1 was NM_201417. For upregulating the expression of RXFP1 in ADSCs, 2 lentiviruses carrying the CRISPRa system were purchased to transfect cells (GenecChem, Shanghai, China). One encoded dCas9-VP64, and the other contained RXFP1-targeting sgRNA or scrambled sgRNA. The sequences of sgRNA were as follows: (1) sgRNA1 (AATTAATGAAAGATAAAACG); (2) sgRNA2 (CTGCAGTCTTAGCAGCTATA); (3) sgRNA3 (GAGTCGCGCACAGCTCACAG). The multiplicity of infection was explored and finally confirmed to be 90.

### 2.3. Animals

The design of our study was approved by the Committee on Animal Experiments of Tongji Hospital, Tongji Medical College, Huazhong University of Science and Technology (TJH-201910005).

A total of 55 male SD rats (8 weeks old) were included in this study, which were from the Laboratory Animal Center of Tongji Medical College, Wuhan, China. Before the formal experiment, all rats received a one-week adaptation (free access to water and food, suitable temperature). The normal sexual function of all animals was confirmed by paired experiments [15,24,25]. Fasting blood glucose and body weight were recorded throughout the experiment.

Streptozotocin (STZ; Sigma-Aldrich, St Louis, MO, USA; 1%) dissolved in its vehicle (0.1 mol/L citrate phosphate buffer; pH 4.2) was used to model diabetes in rats. Intraperitoneal injection of STZ (60 mg/kg) was performed on 47 rats, while the vehicle was injected intraperitoneally into the remaining 8 rats. Successful diabetes models only referred to rats with fasting blood glucose levels greater than 16.7 mmol/L after 72 h.

After eight weeks, the apomorphine (APO) experiment (100 μg/kg; subcutaneous injection) was used to detect erectile function [26]. The number of erections in rats was observed and recorded 30 min after administration. Rats without penile erection (APO-negative rats) were true DMED rats and were used in subsequent experiments. Finally, 24 DMED rats were randomly divided into 3 groups (*n* = 8 per group) and received intracavernous injection: rats without treatment (phosphate-buffered saline; 100μL; DMED group), rats treated with sgRNA-NC ADSCs (1 × 106 cells /100 μL; ADSCs group), and rats treated with RXFP1-ADSCs (1 × 106 cells /100 μL; RXFP1-ADSCs group). The experimental design is presented in Figure S1.

### 2.4. Evaluation of Erectile Function

After 4 weeks, we performed the APO experiment again on all rats. Then, the intracavernous pressure (ICP) and arterial pressure were measured under electrical stimulation of the cavernous nerve (15 Hz; 5.0 V; 1 min) to more intuitively assess erectile function. After the evaluation was completed, the corpus cavernosum was divided into sections and stored at −80 °C and in 4% paraformaldehyde, respectively. Specimens were subsequently prepared as frozen sections and paraffin sections.

### 2.5. Western Blot

The protein lysates (RIPA buffer; Boster) of corpus cavernosum and ADSCs were obtained to detect the expressions of related proteins. After the quantification of protein by the BCA assay (Boster), protein samples would be subjected to electrophoresis, transmembrane and incubation with antibodies. The final visualization of the bands was achieved using a ChemiDocTM MP Image System (Bio-Rad Laboratories, Hercules, CA, USA). The details of the primary antibodies used are listed in Table S1.

### 2.6. Quantitative Reverse-Transcription PCR

The total RNA of ADSCs was isolated with the RNA extraction reagent (Servicebio, Wuhan, China). Quantitative reverse-transcription PCR (qRT-PCR) was performed after the synthesis of cDNA (Yeasen). The primer sequences are as follows: RXFP1, 5’-GCCAAACTCAAGTCTCTCAGCC-3’ (sense), 5’-GAGAGGAGATCCCATCCGTG-3’ (antisense) and β-actin, 5’-CTTCAACACCCCAGCATGT-3’ (sense), 5’-AGTGGTACGACCAGAGGCATACA-3’ (antisense). The method of 2-ΔΔCT was employed to perform quantitative analysis.

### 2.7. Histological Alteration

Paraffin sections (4 μm thickness) were prepared for the procedure of immunohistochemistry (IHC) and immunofluorescence (IF). The area and intensity of the positive region reflected the distribution and expression levels of the target molecules. The details of the antibodies used are listed in Table S1. The normal goat IgG (1: 200; GB23303; Servicebio) was used as a negative control in IHC and presented in Figure S2.

Masson trichrome staining was performed using paraffin sections. The red and blue areas represented the component of smooth muscle and collagen, respectively. Resorcinol-fuchsin staining was also performed for the detection of elastin levels in the penis. The purple-black part represented elastic fibers, the red part represented collagen fibers, and the yellow part in the background referred to other components. The above indicators could partly reflect the level of fibrosis in the penis.

Terminal deoxynucleotidyl transferase-mediated nick end labeling (TUNEL) staining (Beyotime Biotechnology, Shanghai, China) was performed to detect the apoptotic degree in the penis. When genomic DNA was broken, the exposed 3’-OH could be bound by the probe. It was followed by co-incubation with diaminobenzidine for color development.

Frozen sections (10 μm thickness) were prepared to detect reactive oxygen species (ROS). The probe of Dihydroethidium (DHE; Beyotime Biotechnology) was incubated with tissue slices, and the intensity of red fluorescence could reflect the level of ROS under a fluorescence microscope.

### 2.8. Detection of Special Substances

Tissue homogenate was first prepared from the frozen penis with reference to the respective protocol. The protein concentrations of each sample needed to be determined by the BCA assay (Boster) before the detection of subsequent indicators.

Considering that human RLX-2 is equivalent to rodent RLX-1 [27], the ELISA kits of RLX-1 (BANGYI, Shanghai, China), VEGF, bFGF and cyclic guanosine monophosphate (cGMP; Mbbiology, Jiangsu, China) were used to detect respective target molecules. Different concentrations of standards and 10 uL samples were added to the sample wells coated with relevant antigens. Subsequently, the reaction system containing other reagents was incubated at 37 °C. Absorbance was read at a wavelength of 450 nm using a microplate reader.

A total NO assay kit (Beyotime Biotechnology) was used to detect nitric oxide (NO). The standard was diluted to different gradient concentrations. Then, 60 uL samples, or standards of different concentrations, and other reaction components, were added to each well of 96-well plates. After incubation at 37 °C, color development was recorded with a microplate reader at 540 nm absorbency.

For oxidative activity, malondialdehyde (MDA) and superoxide dismutase (SOD) were chosen to be examined with respective test kits (Beyotime Biotechnology). In addition to the sample’s reaction, the standard and the control reaction also needed to be carried out. For the detection of SOD, the absorbance at a wavelength of 450 nm was detected after incubation at 37 °C. For the detection of MDA, the absorbance at a wavelength of 532 nm was caught after a water bath at 100 °C.

In addition, we normalized the above results using the respective protein concentrations. The final results reflected the amount of substance to be measured per unit mass of penile tissue.

### 2.9. Statistical Analyses

The data were expressed as the mean ± standard deviation and analyzed using GraphPad Prism version 8.0 (GraphPad Software, San Diego, CA, USA). The Shapiro–Wilk test was used to determine normal distribution. For normally distributed data, we used one-way ANOVA analysis and Tukey’s test for multiple comparisons. For non-normally distributed data, we used the Kruskal–Wallis test and Dunn’s test for multiple comparisons. A *p*-value < 0.05 indicated that the difference was statistically significant.

## 3. Results

### 3.1. Preparation and Transfection of ADSCs

**Figure 1:**
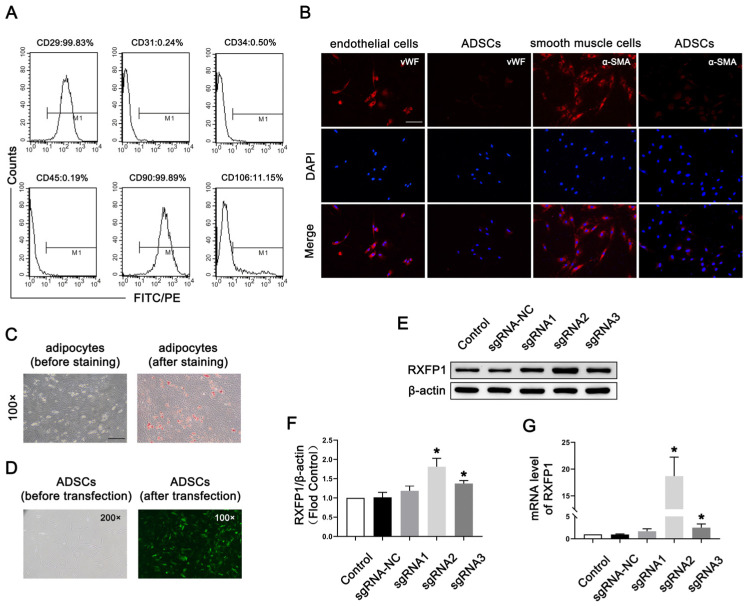
Isolation and transfection of ADSCs. (**A**) Representative images of flow cytometry of ADSCs for identification. (**B**) Representative immunofluorescence (×200, bars = 100 µm) of vWF and α-SMA in ADSCs after induced differentiation to endothelial and smooth muscle cells. (**C**) Representative images of Oil-red-O staining (×100, bars = 200 µm) of ADSCs after induced differentiation to adipocytes. (**D**) Representative images of ADSCs before and after transfection. Representative immunoblot (**E**) and semi-quantification (**F**) of RXFP1 of ADSCs in different groups. (**G**) The mRNA expression levels of RXFP1 in ADSCs in different groups; *n* = 4 for each group. * *p* < 0.05 vs. the control group. ADSCs = adipose-derived stem cells; sgRNA = single guide RNA; RXFP1 = relaxin family peptide receptor 1.

As shown in Figure 1A, the surface markers of most ADSCs at passage 4 appeared as follows: CD29 (+), CD31 (−), CD34 (−), CD45 (−), CD90 (+) and CD106 (−). The above indicators showed that the primary cells we isolated from adipose were mesenchymal stem cells. The results of IF were positive for vWF and α-SMA, suggesting that ADSCs could differentiate into endothelial and smooth muscle cells (Figure 1B). The results of Oil-red-O staining were also positive, meaning that ADSCs could differentiate into adipocytes (Figure 1C).

Lentiviruses carrying the CRISPRa system were successfully transfected into ADSCs (Figure 1D). The results of Western blotting (WB) and qRT-PCR revealed that our genetic modification of ADSCs was effective (Figure 1E–G). The expression of RXFP1 increased in the two specific RXFP1-targeting sgRNA groups (sgRNA2 and sgRNA3), and the sgRNA2 group had the most noticeable effect. As a result, we applied the ADSCs of the sgRNA2 group to the follow-up animal experiments.

### 3.2. Metabolic and Physiological Parameters

**Figure 2:**
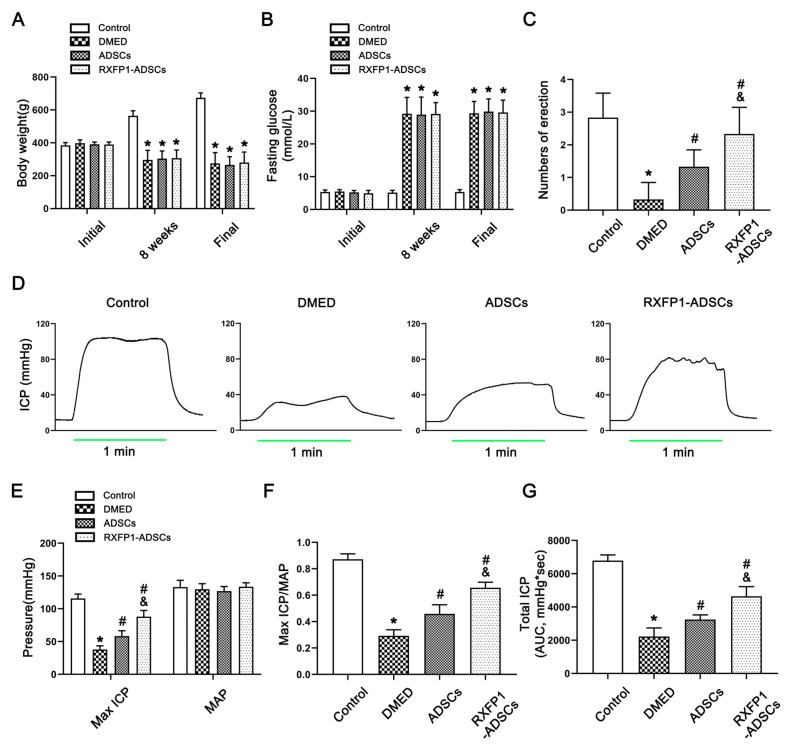
Assessment of metabolic and physiological parameters of rats. Body weight (**A**) and fasting blood glucose (**B**) in different groups during the trial; *n* = 8 for each group. (**C**) The result of the APO experiment in different groups; *n* = 6 for each group. Representative recordings (**D**) and semi-quantification (**E**) of ICP and AP in different groups under electrical stimulation (15 Hz; 5.0 V; 1 min). The result of Max ICP/MAP (**F**) and total ICP (**G**) in different groups; *n* = 5 for each group. * *p* < 0.05 vs. the control group; # *p* < 0.05 vs. the DMED group; & *p* < 0.05 vs. the ADSCs group. DMED = diabetes mellitus-induced erectile dysfunction; ADSCs = adipose-derived stem cells; RXFP1 = relaxin family peptide receptor 1; APO = apomorphine; ICP = intracavernous pressure; AP = arterial pressure; MAP = mean arterial pressure; AUC = area under the curve.

All rats’ body weight and fasting blood glucose were similar at the start (all *p* > 0.05; Figure 2A,B). Eight weeks after the induction of the diabetes model, the body weight decreased, and the blood glucose increased in DM rats compared with the control group (both *p* < 0.05). Additionally, this trend did not change 4 weeks after ADSC transplantation. These results indicated that the construction of DM rats was successful.

The APO experiment showed that the number of erections was lowest in the DMED group and increased in the treatment groups (all *p* > 0.05; Figure 2C). In addition, we also measured the arterial pressure and ICP to assess erectile function in each group (Figure 2D–G). Under the condition that the mean arterial pressure (MAP) was relatively constant, the maximum ICP/MAP and total ICP (AUC, area under the curve) were decreased in the DMED group and increased in the treatment groups (all *p* < 0.05), and the RXFP1-ADSC group was more significant.

**Figure 3:**
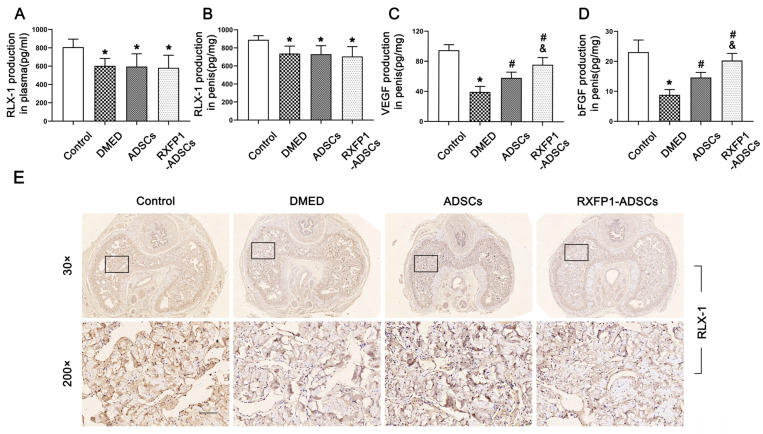
Detection of RLX-1, VEGF and bFGF levels in vivo. Levels of RLX-1 in plasma (**A**) and penis (**B**) for each group; *n* = 6 for each group. Levels of VEGF (**C**) and bFGF (**D**) in the penis for each group; *n* = 4 for each group. (**E**) Representative immunohistochemistry (×30 and ×200, bars = 100 µm) of RLX-1 in penis for each group. * *p* < 0.05 vs. the control group; # *p* < 0.05 vs. the DMED group; & *p* < 0.05 vs. the ADSCs group. RLX-1 = relaxin-1; DMED = diabetes mellitus-induced erectile dysfunction; ADSCs = adipose-derived stem cells; RXFP1 = relaxin family peptide receptor 1.

The ELISA assays and IHC indicated the presence of RLX-1 in plasma and penile tissue, and its levels in DMED rats were slightly reduced compared to the control group and did not change after ADSC transplantation (*p* < 0.05; Figure 3A,B,E). The concentrations of VEGF and bFGF declined in the DMED group and recovered to some extent after ADSC transplantation, contrasted with the control group (all *p* < 0.05; Figure 3C,D). Moreover, RXFP1 was expressed in the corpus cavernosum sinus, and the level in the DMED group was decreased compared with the control group (Figure S3).

### 3.3. Transplantation of ADSCs Inhibited Oxidative Stress Damage in Penile Tissue

**Figure 4:**
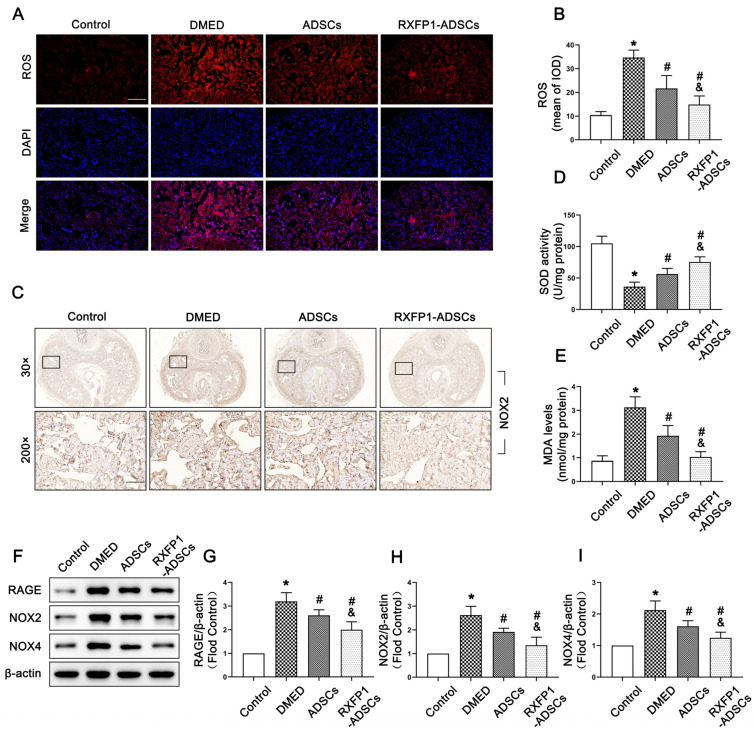
Effects of ADSCs transplantation on reducing oxidative stress in penile tissue. Representative fluorescence (×100, bars = 200 µm) (**A**) and semi-quantification (**B**) of ROS in the penis for each group; *n* = 5 for each group. (**C**) Representative immunohistochemistry (×30 and ×200, bars = 100 µm) of NOX2 in different groups. Levels of SOD (**D**) and MDA (**E**) in different groups. Representative immunoblot (**F**) and semi-quantification (**G**–**I**) of RGAE, NOX2 and NOX4 in different groups; *n* = 4 for each group. * *p* < 0.05 vs. the control group; # *p* < 0.05 vs. the DMED group; & *p* < 0.05 vs. the ADSCs group. ROS = reactive oxygen species; DMED = diabetes mellitus-induced erectile dysfunction; ADSCs = adipose-derived stem cells; RXFP1 = relaxin family peptide receptor 1; SOD = superoxide dismutase; MDA = malondialdehyde.

The levels of ROS and MDA were chosen to be examined for detecting the activity of oxidative stress. As shown in Figure 4A,B,E, the levels of ROS and MDA in DMED rats were higher compared with the control group and decreased after treatment of ADSCs, specifically RFXP1-ADSCs (all *p* < 0.05). Contrarily, SOD, an important antioxidant, exhibited opposite changes in penile tissue (*p* < 0.05; Figure 4D). The RAGE and NADPH oxidases, including NOX2 and NOX4, are regarded to be critical in the process of oxidative stress. The results of WB and IHC suggested that the expressions of RAGE, NOX2 and NOX4 were highest in the DMED group and inhibited to a certain extent after ADSC transplantation (all *p* < 0.05; Figure 4C,F–I).

### 3.4. Transplantation of ADSCs Regulated the NO/cGMP and RhoA/ROCK Pathway in Rats

**Figure 5:**
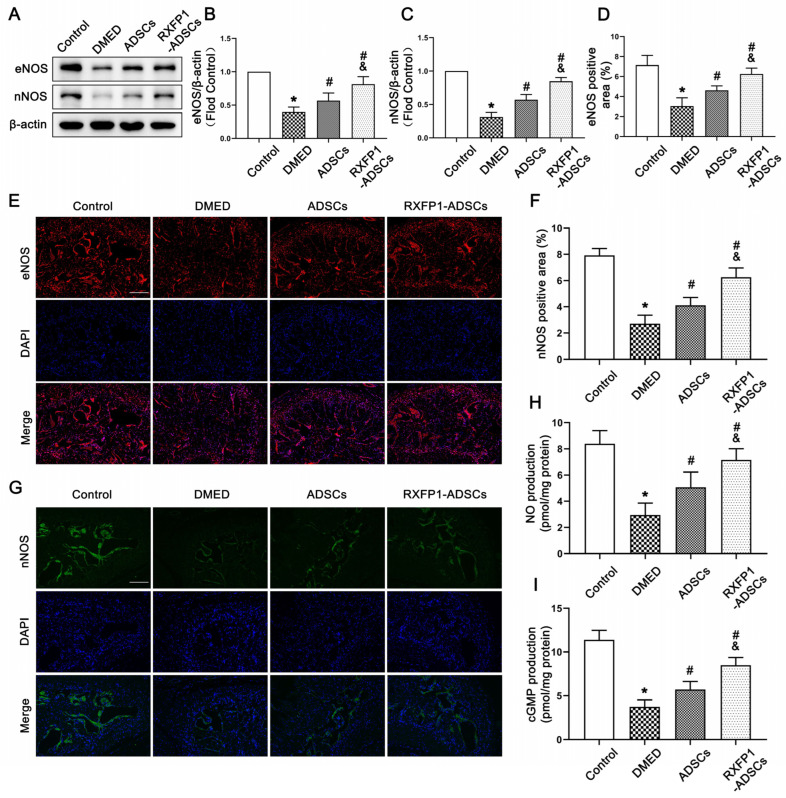
Effects of ADSCs transplantation on regulating the NO/cGMP pathway in rats. Representative immunoblot (**A**) and semi-quantification (**B**,**C**) of eNOS and nNOS in the penis for each group; *n* = 5 for each group. Representative immunofluorescence (×100, bars = 200 µm) (**E**,**G**) and semi-quantification (**D**,**F**) of eNOS and nNOS in different groups. The concentration of NO (**H**) and cGMP (**I**) in different groups; *n* = 4 for each group. * *p* < 0.05 vs. the control group; # *p* < 0.05 vs. the DMED group; & *p* < 0.05 vs. the ADSCs group. DMED = diabetes mellitus-induced erectile dysfunction; ADSCs = adipose-derived stem cells; RXFP1 = relaxin family peptide receptor 1; NO = nitric oxide; cGMP = cyclic guanosine monophosphate.

The results of WB and IF demonstrated that the expressions of eNOS and nNOS were downregulated under long-term diabetes (both *p* < 0.05; Figure 5A–G). After ADSC transplantation, the expressions of the above two molecules were upregulated but lower than the control group (all *p* < 0.05). The alteration of the NO/cGMP pathway, as the downstream pathway of eNOS and nNOS, was consistent with the above two molecules (Figure 5H,I).

**Figure 6:**
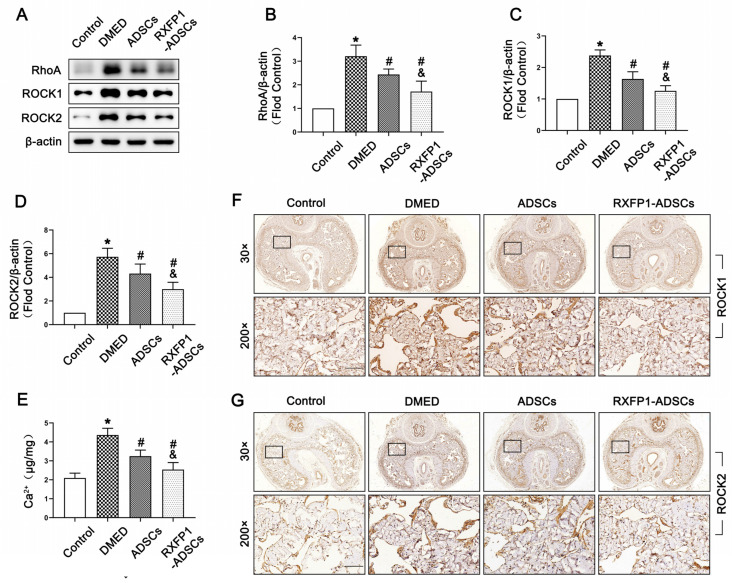
Effects of ADSCs transplantation on regulating the RhoA/ROCK pathway in rats. Representative immunoblot (**A**) and semi-quantification (**B**–**D**) of RhoA, ROCK1 and ROCK2 in the penis for each group. (**E**) The concentration of Ca^2+^ in different groups. *n* = 4 for each group. Representative immunohistochemistry (×30 and ×200, bars = 100 µm) of ROCK1 (**F**) and ROCK2 (**G**) in different groups. * *p* < 0.05 vs. the control group; # *p* < 0.05 vs. the DMED group; & *p* < 0.05 vs. the ADSCs group. DMED = diabetes mellitus-induced erectile dysfunction; ADSCs = adipose-derived stem cells; RXFP1 = relaxin family peptide receptor 1.

On the contrary, we found the highest expression of RhoA, ROCK1 and ROCK2 in the DMED group (all *p* < 0.05; Figure 6A–D,F,G). The concentration of Ca^2+^ was also significantly reinforced in DMED rats compared with the control rats (*p* < 0.05; Figure 6E). Moreover, these four indicators were dampened after treatment of ADSCs, in which the more significant effect was found in the RXFP1-ADSCs group.

### 3.5. Transplantation of ADSCs Adjusted Apoptosis In Vivo

**Figure 7:**
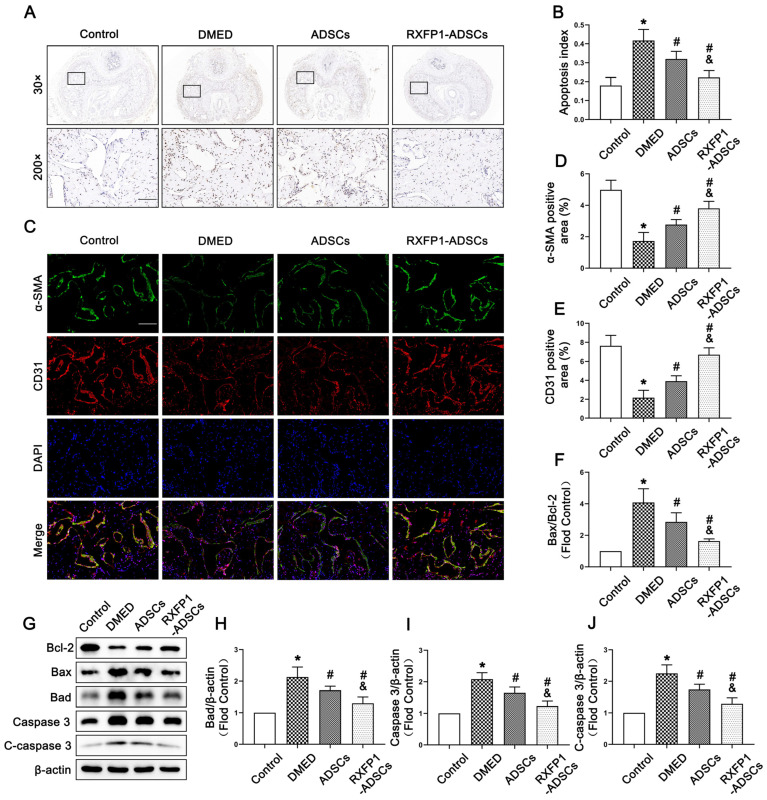
Effects of ADSCs transplantation on adjusting apoptosis in vivo. (**A**) Representative images of TUNEL staining (×30 and ×200, bars = 100 µm) (**A**) and apoptosis index (**B**) in the penis for each group. Representative immunofluorescence (×200, bars = 100 µm) (**C**) and semi-quantification (**D**,**E**) of α-SMA and CD31 in different groups. Representative immunoblot (**G**) and semi-quantification (**F**,**H**–**J**) of Bcl-2, Bax, Bad, Caspase 3 and C-caspase 3 in different groups. *n* = 4 for each group. * *p* < 0.05 vs. the control group; # *p* < 0.05 vs. the DMED group; & *p* < 0.05 vs. the ADSCs group. DMED = diabetes mellitus-induced erectile dysfunction; ADSCs = adipose-derived stem cells; RXFP1 = relaxin family peptide receptor 1; TUNEL = Terminal deoxynucleotidyl transferase-mediated nick end labeling staining; C-caspase 3 = cleaved Caspase 3.

The result of TUNEL staining indicated that the apoptosis index in DMED rats was highest among the four groups and declined after treatment of ADSCs but was still lower than the control group (all *p* < 0.05; Figure 7A,B). The expression of α-SMA and CD31 (the markers of smooth muscle cells and endothelial cells, which are the primary effector cells of the corpus cavernosum) also conformed to the above trend (Figure 7C–E). Furthermore, we found that the ratio of Bax to Bcl-2, the expression of Caspase 3 and cleaved Caspase 3 (C-caspase 3) could be recovered to different degrees under the effects of ADSCs and RXFP1-ADSCs (all *p* < 0.05; Figure 7F–J). The above results suggested that ADSCs could alleviate apoptosis in vivo, and RXFP1-ADSCs were more significant.

### 3.6. Transplantation of ADSCs Reduced Fibrosis in the Corpus Cavernosum

**Figure 8:**
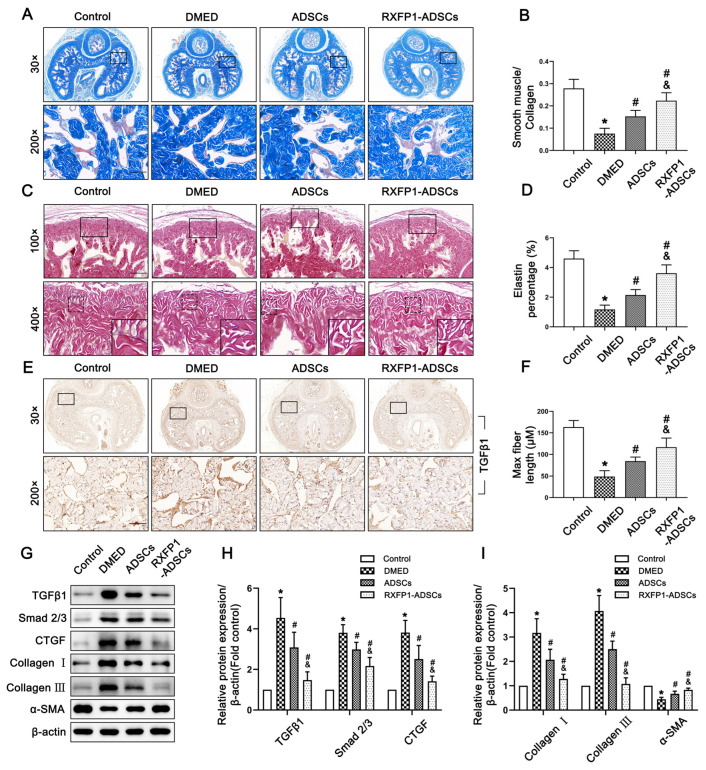
Effects of ADSCs transplantation on reducing fibrosis in the corpus cavernosum of rats. Representative images of Masson trichrome staining (×30 and ×200, bars = 100 µm) (**A**) and semi-quantification (**B**) in penis for each group. Representative images of resorcinol-fuchsin staining (×100, bars = 200 µm) (**C**) and semi-quantification (**D**,**F**) in different groups. (**E**) Representative immunohistochemistry (×30 and ×200, bars = 100 µm) of TGFβ1 in different groups. Representative immunoblot (**G**) and semi-quantification (**H**,**I**) of TGFβ1, Smad 2/3, CTGF, Collagen I, Collagen III and α-SMA in different groups. *n* = 4 for each group. * *p* < 0.05 vs. the control group; # *p* < 0.05 vs. the DMED group; & *p* < 0.05 vs. the ADSCs group. DMED = diabetes mellitus-induced erectile dysfunction; ADSCs = adipose-derived stem cells; RXFP1 = relaxin family peptide receptor 1.

Masson trichrome staining and resorcinol-fuchsin staining are both indicators reflecting the level of fibrosis. The ratio of smooth muscle to collagen significantly decreased in DMED rats and increased to a certain extent in the treatment group (all *p* < 0.05; Figure 8A,B). However, the detection of elastic fibers showed the opposite result. The elastin percentage and maximum elastic fiber length in the DMED group were the lowest among the four groups (both *p* < 0.05; Figure 8C,D,F). Furthermore, WB also verified the above results. The TGFβ1/Smad 2/3/CTGF pathway and the expression of Collagen I, Collagen III and α-SMA of DMED rats all showed a trend opposite to those of control rats and recovered to varying degrees after ADSC transplantation (all *p* < 0.05; Figure 8E,G–I).

## 4. Discussion

As a metabolic abnormality, DM has a high incidence worldwide and is one of the important causes of ED [1,2]. Due to the limited effects of therapies for DMED, it has troubled a large number of patients and medical workers around the world. Transplantation of ADSCs is a promising treatment for DMED. Given the solid regulatory capacity of RXFP1 in pathological settings, we activated the expression of RXFP1 in ADSCs via CRISPRa to achieve a better therapeutic effect on DMED. In the present study, we successfully expressed high levels of RXFP1 in ADSCs and performed intracavernous injections of them in DMED rats. The results showed that RXFP1-ADSC transplantation partially improved erectile function and had more potent antioxidant, anti-apoptotic and anti-fibrotic abilities than NC ADSCs. In addition, RXFP1-ADSCs could better regulate the content and function of endothelial cells and smooth muscle cells.

Multi-lineage differentiation ability and paracrine factors of mesenchymal stem cells are the two keys to treating ED [9]. The mechanism mainly includes the directed differentiation function of ADSCs into cavernous endothelial cells and smooth muscle cells, as well as a large number of paracrine cytokines, such as VEGF and bFGF. This maintains the number and function of essential cells in the corpus cavernosum and resists pathological changes [28,29,30]. Although the efficiency of differentiation to endothelial or smooth muscle cells is controversial, we cannot yet wholly deny that this mechanism is involved in the treatment of ED with mesenchymal stem cells [30]. In our study, we confirmed in in vitro experiments that ADSCs could differentiate into endothelial and smooth muscle cells, which were the primary effector cells of the corpus cavernosum and participated in the physiological erection. The content and function of endothelial and smooth muscle cells in rats also increased after ADSC transplantation, which may be a combined result of the differentiation of ADSCs and decreased levels of apoptosis. Increased levels of VEGF and bFGF in the penis suggested that paracrine trophic factors of ADSCs promoted the restoration of erectile function. In addition, recent studies have pointed out that exosomes secreted by mesenchymal stem cells also play a protective role in erectile function. Chen et al. and Zhu et al. proposed that ADSC-derived exosomes could independently ameliorate ED in diabetic rats [31,32]. This may be a new option for future cell-free therapy in DMED. PGI2 also plays a protective role in improving penile erection and can be mediated by RLX-1 [33,34]. The activation of RLX-1-RXFP1 signaling in ADSCs may also increase the secretion of PGI2 to upregulate the cAMP pathway in the surrounding corpus cavernous.

Efficacy after transplantation of mesenchymal stem cells is often limited due to various factors, such as (1) shortened cell lifespan resulting from multiple expansion of cells in vitro or (2) a hostile microenvironment at the transplant site. To improve therapeutic efficacy, genetic modification is performed as one of the logical options to enhance cell survival and function [9,35,36]. The activation of RXFP1 may play a protective role in cardiovascular disease [37]. RXFP1 is also involved in the functional exercise of mesenchymal stem cells and increases the therapeutic effect of adult cells [13,14]. Therefore, we reasonably inferred that the upregulation of RXFP1 may also promote the efficacy of ADSCs in lesions. For the purpose of overexpressing RXFP1 in ADSCs, CRISPRa seems to be a good choice. Compared with other existing gene-editing strategies, CRISPRa offers the following advantages: (1) it induces low off-target effects; (2) it is independent of the target gene size and can activate different genes at the same time; (3) it can simultaneously up- or downregulate different genes in target cells; and (4) it is a mutation-independent therapeutic strategy [38]. Moreover, CRISPRa has been successfully used to genetically modify several stem cells, including ADSCs, BMSCs, induced pluripotent stem cells and other stem cells, to achieve the goal of tissue regeneration or disease treatment [19,39,40]. Given the above theoretical basis, we upregulated RXFP1 expression in ADSCs via CRISPRa, followed by intracavernous injection in DMED rats. Our data showed that ADSCs could increase erectile function through multiple mechanisms, and the effect of RXFP1-ADSC was more potent than that of NC ADSCs. The above results proved that RXFP1 enhanced the therapeutic effect of ADSCs in DMED.

Although DMED is a complex pathological process with multiple factors, oxidative stress plays a critical role. Oxidative stress refers to the imbalance between ROS production and the endogenous antioxidant system. Advanced glycation end-products (AGEs) aggravate ROS production and have an exceptional contribution to oxidative stress, while diabetes is the disease most conducive to the formation of AGEs [41]. Oxidative stress plays a vital role in microvascular injury through various factors [42]. Since the corpus cavernosum happens to be part of the whole body’s microvessels, numerous studies have been conducted to ameliorate DMED by reducing oxidative stress [43,44]. The data of our in vivo experiments also revealed the increase of ROS and the decrease of SOD in the penis of diabetic rats, indicating the high level of oxidative stress in DMED. As an essential source of oxidative stress, the activity of NADPH was also confirmed to be enhancive in DMED. The above pathological changes were all reversed to some extent after ADSC transplantation, especially in the case of RXFP1-ADSCs.

Long-term diabetes can also lead to other dysfunctions of the corpus cavernosum, including increased apoptosis and fibrosis, which promote the occurrence or progression of DMED [45,46]. The intracavernous injection of mesenchymal stem cells has been shown to improve erectile function through anti-apoptosis and anti-fibrosis effects [45,47]. The aforementioned protective effects were indeed found in DMED rats after ADSC transplantation in this study, and the upregulation of RXFP1 expression amplified the benefits of ADSCs. The imbalance of smooth muscle relaxation and contraction also participates in the development of DMED. NO is the primary substance that promotes the relaxation of the smooth muscle of the corpus cavernosum and is produced by two enzymes, eNOS and nNOS. The RhoA/ROCK pathway is an important signaling pathway that regulates smooth muscle contraction. Imbalance in the NO/cGMP and RhoA/ROCK pathways also aggravates DMED [44,48,49]. The present study suggested the imbalance of the NO/cGMP and RhoA/ROCK pathways appeared in DMED rats, and ADSC transplantation regulated these two pathways to normal levels. Moreover, oxidative stress can induce or exacerbate the above-mentioned pathological changes, including apoptosis, fibrosis and smooth muscle dysfunction. Reduced levels of oxidative stress contribute to a multifactorial improvement of erectile function in diabetic rats [44,45,48].

**Figure 9:**
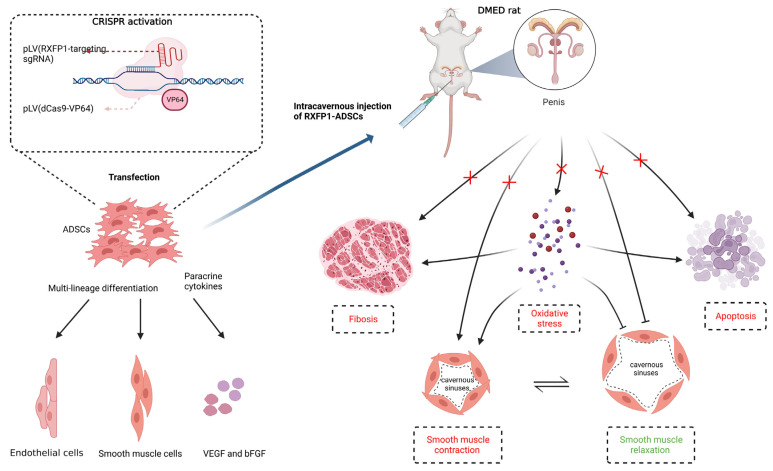
Potential mechanism of ADSCs Transplantation in the treatment of DMED. Transplantation of ADSCs improves erectile function in DMED rats through differentiating into functional cells and secreting cytokines such as VEGF and bFGF. Regulation of oxidative stress, apoptosis and fibrosis are involved in this process. The normalization of endothelial and smooth muscle cells′ function also follows the above alteration. Moreover, reduced level of oxidative stress greatly benefits the improvement of the pathological environment in the penis. Created with BioRender.com. CRISPR = clustered regularly interspaced short palindromic repeats; sgRNA = single guide RNA; dCas9 = catalytically inactive CRISPR-associated protein 9; RXFP1 = relaxin family peptide receptor 1; ADSCs = adipose-derived stem cells; DMED = diabetes mellitus-induced erectile dysfunction.

Based on the above results, we generalized the possible underlying mechanism of RXFP1-ADSCs in ameliorating DMED (Figure 9). The long-term chronic high-glucose environment in the local tissue of the penis induces an increase in oxidative stress. Histiocytic apoptosis and fibrosis also occur in damaged tissues. At the same time, the content and function of endothelial cells and smooth muscle cells in the penile cavernous sinuses decreased under the harsh local microenvironment. Oxidative stress could, in turn, aggravate levels of apoptosis, fibrosis and endothelial cell and smooth muscle cell dysfunction. The abovementioned multiple complex factors together lead to the decline of erectile function. Transplantation of ADSCs can reverse these pathological changes to a certain extent by differentiating into functional cells and secreting cytokines such as VEGF and bFGF. Moreover, RXFP1-ADSCs can further amplify these beneficial effects and further improve erectile function.

Limitations still existed in our study. Firstly, we did not directly test the survival or resident capacity of ADSCs in high-glucose environments in vitro or in diabetic animals. Moreover, the therapeutic effect of mesenchymal stem-cell-derived exosomes on DMED has been confirmed, which was not involved in our experiments. Finally, other subtypes of relaxin and receptors for relaxin also exist besides those explored in this study. Considering there may be cross-effects among them, other subtypes still merit exploration. The above limitations need to be further explored in our future research.

## 5. Conclusions

RXFP-ADSCs were confirmed to alleviate erectile dysfunction in diabetic rats by increasing the proliferative and paracrine capacity of ADSCs. The possible therapeutic effects of RXFP1-ADSCs were associated with the regulation of oxidative stress, apoptosis and fibrosis. The normalization of endothelial and smooth muscle cell function was also involved in this process. Our research provided new ideas for the application of mesenchymal stem cells and contributed to the development of regenerative and translational medicine.

## Data Availability

The data presented in this study are available on request from the corresponding author.

## Supplementary Materials

The following supporting information can be downloaded at: https://www.mdpi.com/article/10.3390/antiox12010171/s1, Figure S1: Flow Diagram of Experimental Design; Figure S2: Negative controls of immunohistochemistry in each group; Figure S3: The expression of RXFP1 in the penis of rats; Table S1: The details of the antibodies used in the study.
