# Correction: Sun et al. Engineered Adipose-Derived Stem Cells Overexpressing RXFP1 via CRISPR Activation Ameliorate Erectile Dysfunction in Diabetic Rats. *Antioxidants* 2023, *12*, 171

**DOI:** 10.3390/antiox15030304

**Published:** 2026-02-28

**Authors:** Taotao Sun, Wenchao Xu, Bocheng Tu, Tao Wang, Jihong Liu, Kang Liu, Yang Luan

**Affiliations:** 1 Department of Urology, Tongji Hospital, Tongji Medical College, Huazhong University of Science and Technology, Wuhan 430030, China; 2 Institute of Urology, Tongji Hospital, Tongji Medical College, Huazhong University of Science and Technology, Wuhan 430030, China

In the original publication [1], Figure 1C. Specifically, the authors inadvertently attached the wrong picture for Figure 1C. This error occurred during the figure preparation process and was not detected prior to publication.

The corrected Figure 1 and its legend appears below. The authors state that the scientific conclusions are unaffected. This correction was approved by the Academic Editor. The original publication has also been updated.

**Figure 1:**
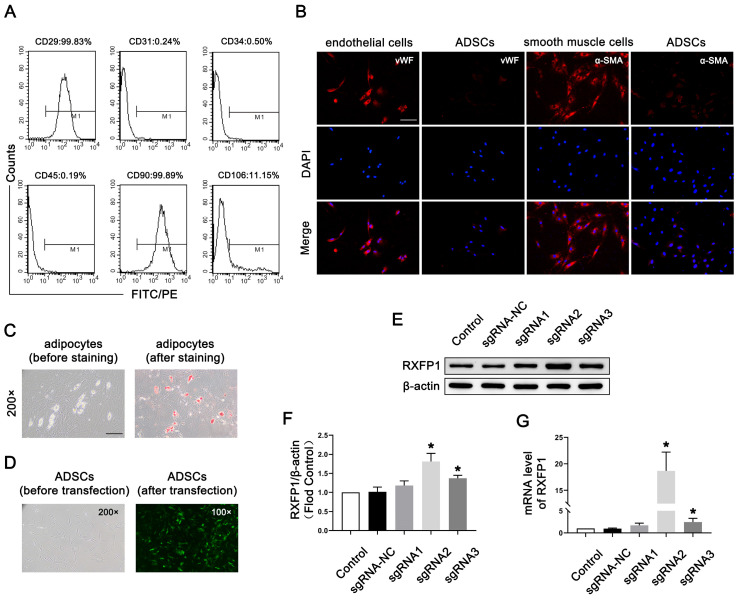
Isolation and transfection of ADSCs. (**A**) Representative images of flow cytometry of ADSCs for identification. (**B**) Representative immunofluorescence (×200, bars = 100 µm) of vWF and α-SMA in ADSCs after induced differentiation to endothelial and smooth muscle cells. (**C**) Representative images of Oil-red-O staining (×200, bars = 100 µm) of ADSCs after induced differentiation to adipocytes. (**D**) Representative images of ADSCs before and after transfection. Representative immunoblot (**E**) and semi-quantification (**F**) of RXFP1 of ADSCs in different groups. (**G**) The mRNA expression levels of RXFP1 in ADSCs in different groups; *n* = 4 for each group. * *p* < 0.05 vs. the control group. ADSCs = adipose-derived stem cells; sgRNA = single guide RNA; RXFP1 = relaxin family peptide receptor 1.
