# Supplementary material for: Engineered Adipose-Derived Stem Cells Overexpressing RXFP1 via CRISPR Activation Ameliorate Erectile Dysfunction in Diabetic Rats
Source: Antioxidants (Basel). 2023 Jan 11;12(1):171. doi: 10.3390/antiox12010171 (PMC9854730; doi:10.3390/antiox12010171)
Supplement: Supplementary file 1 [file antioxidants-12-00171-s001.zip › antioxidants-1990014-supplementary.pdf]

Supplementary Materials

Supplementary Table S1. The details of the antibodies used in the study.

	Name	Abbreviation	concentration	source
FC	anti-CD29	CD29	1:200	11-0291-80; Invitrogen, Rockford, IL, USA
	anti-CD31	CD31	1:200	555027; BD Biosciences, San Diego, CA, USA
	anti-CD34	CD34	1:200	sc-7324; Santa Cruz Biotechnology, Dallas, Texas, USA
	anti-CD45	CD45	1:200	11-0461-80; Invitrogen
	anti-CD90	CD90	1:200	561973; BD Biosciences
	anti-CD106	CD106	1:200	559229; BD Biosciences
WB	anti-RXFP1	RXFP1	1:1000	44621; Signalway Antibody, MD, USA
	anti-RAGE	RAGE	1:500	16346-1-AP; Proteintech, Wuhan, China
	anti-NOX2	NOX2	1:1000	BM4576; Boster, Wuhan, China
	anti-NOX4	NOX4	1:1000	BM4135; Boster
	anti-eNOS	eNOS	1:1000	ab5589; Abcam, Cambridge, MA, USA
	anti-nNOS	nNOS	1:1000	610309; BD Biosciences
	anti-RhoA	RhoA	1:1000	10749-1-AP; Proteintech
	anti-ROCK1	ROCK1	1:1000	21850-1-AP; Proteintech
	anti-ROCK2	ROCK2	1:1000	21645-1-AP; Proteintech
	anti-Bcl-2	Bcl-2	1:1000	BF9103; Affinity, USA
	anti-Bax	Bax	1:1000	A5131; Bimake, Houston, TX, USA
	anti-Bad	Bad	1:1000	A5147; Bimake
	anti-Caspase-3	Caspase-3	1:1000	A19654; ABclonal, Wuhan, China
	anti-Cleaved Caspase-3	Cleaved Caspase-3	1:1000	A19654; ABclonal
	anti-TGF-β1	TGF-β1	1:1000	21898-1-AP; Proteintech
	anti-Smad2/3	Smad2/3	1:1000	8685S; Cell Signaling Technology, Danvers, MA, USA
	anti-CTGF	CTGF	1:500	23936-1-AP; Proteintech
	anti-Collagen I	Collagen I	1:1000	14695-1-AP; Proteintech
	anti-Collagen III	Collagen III	1:500	M00788; Boster
	anti-α-SMA	α-SMA	1:1000	GB111364; Servicebio, Wuhan, China

	anti-β-actin	β-actin	1:1000	GB11001; Servicebio
IHC	anti-RLX-2	RLX-2	1:50	A6969; ABclonal
	anti-NOX2	NOX2	1:500	19013-1-AP; Proteintech
	anti-ROCK1	ROCK1	1:200	21850-1-AP; Proteintech
	anti-ROCK2	ROCK2	1:200	21645-1-AP; Proteintech
	anti-TGFβ1	TGFβ1	1:300	21898-1-AP; Proteintech
	anti-RXFP1	RXFP1	1:200	18419-1-AP; Proteintech
IF	anti-vWF	vWF	1:100	GB11020; Servicebio
	anti-α-SMA	α-SMA	1:500	GB111364; Servicebio
	anti-eNOS	eNOS	1:100	AF0096; Affinity
	anti-nNOS	nNOS	1:200	GB11145; Servicebio
	anti- CD31	CD31	1:100	GB113151; Servicebio

FC = flow cytometry; WB = western blotting; IHC = immunohistochemistry; IF = immunofluorescence

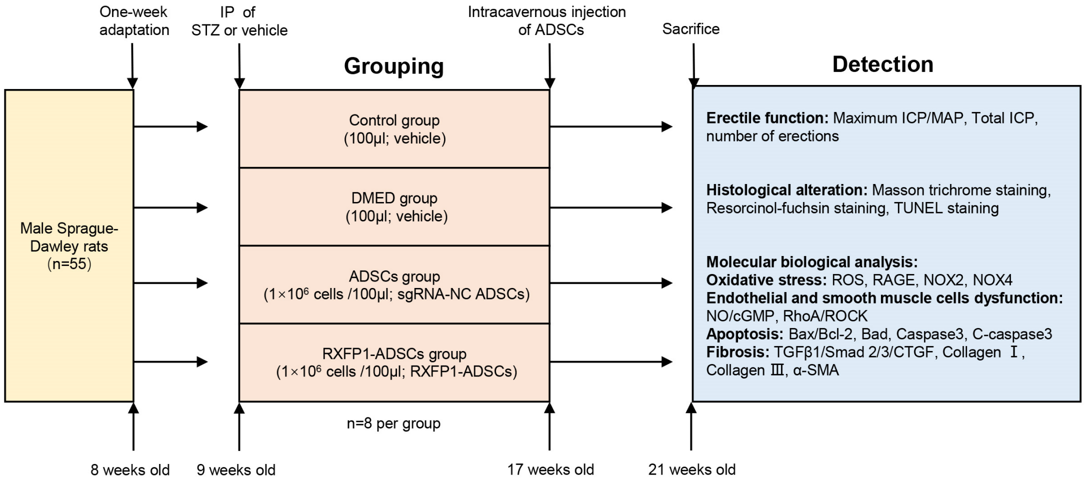

Supplementary Figure S1. Flow Diagram of Experimental Design. ADSCs overexpressing RXFP1 by transfection of lentiviruses with the CRISPR activation system were first prepared and validated. The sequence of sgRNA2 was the most efficient and used for subsequent animal experiments. After one-week adaptation, type 1 diabetic rats were constructed by intraperitoneal injection of 1% STZ (60mg/kg). After 8 weeks, all DMED rats and the remaining 8 normal rats were divided into 4 groups and received intracavernous injection: control group (vehicle), DMED group (vehicle), ADSCs group (1×10^6 cells /100μl; sgRNA-NC ADSCs), RXFP1-ADSCs group (1×10^6 cells /100μl; RXFP1-ADSCs). After another 4 weeks, erectile function was examined in all rats and the corpus cavernosum was collected for subsequent detection for histological and molecular biological analysis. IP = intraperitoneal injection; STZ = streptozotocin; DMED = diabetes mellitus-induced erectile dysfunction; ADSCs = adipose-derived stem cells; sgRNA = single guide RNA; RXFP1 = relaxin family peptide receptor 1.

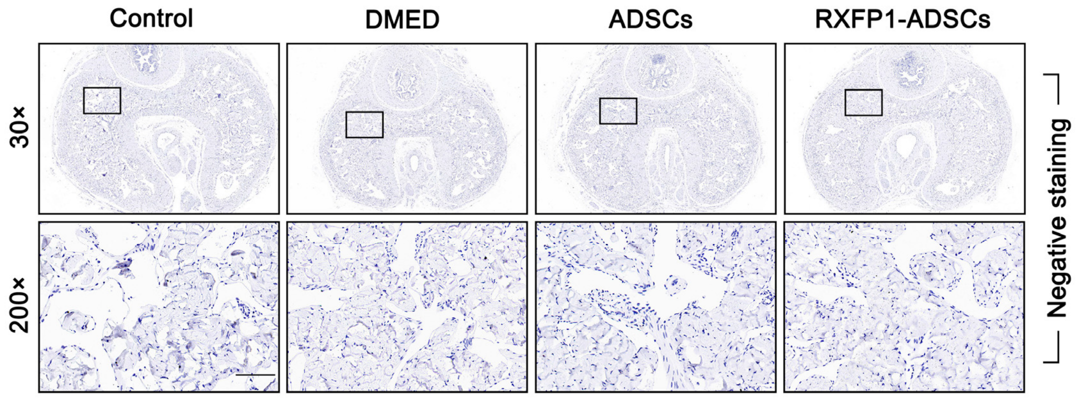

Supplementary Figure S2. Negative controls of immunohistochemistry (× 30 and × 200, bars = 100 μm) in each group. DMED = diabetes mellitus-induced erectile dysfunction; ADSCs = adipose-derived stem cells; RXFP1 = relaxin family peptide receptor 1.

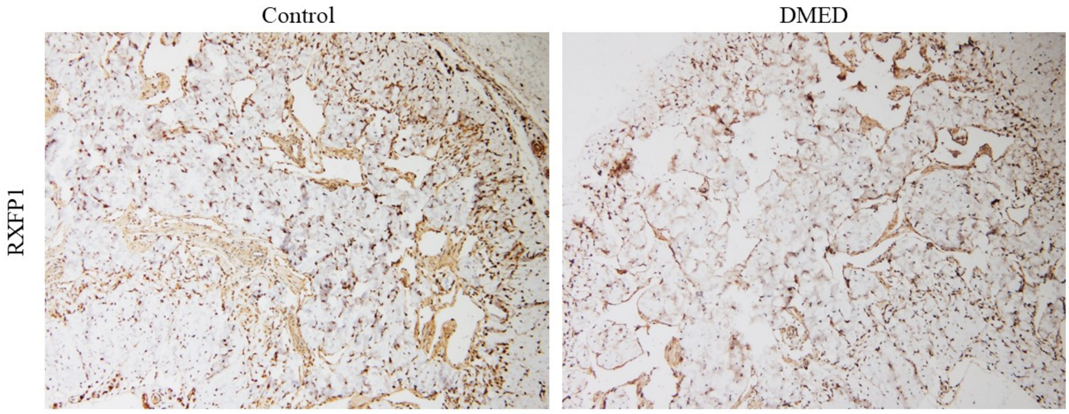

Supplementary Figure S3. The expression of RXFP1 in the penis of rats. Representative immunohistochemistry (× 100) of RXFP1 in penis of the 2 groups. DMED = diabetes mellitus-induced erectile dysfunction; RXFP1 = relaxin family peptide receptor 1.
